# RNA Sequencing for Gene Expression Profiles in Peripheral Blood Mononuclear Cells with Ankylosing Spondylitis RNA

**DOI:** 10.1155/2020/5304578

**Published:** 2020-06-06

**Authors:** Dan Huang, Jian Liu, Yunxiang Cao, Lei Wan, Hui Jiang, Yue Sun, Jianting Wen

**Affiliations:** ^1^Graduate School, Anhui University of Chinese Medicine, No1 Qianjiang Road, Xinzhan District, Hefei, China; ^2^Department of Rheumatology, First Affiliated Hospital of Anhui University of Chinese Medicine, No 117 Meishan Road, Shushan District, Hefei, China; ^3^Rheumatology institute of Anhui Academy Chinese Medicine, No 117 Meishan Road, Shushan District, Hefei, China; ^4^State Administration of Chinese Medicine Preparation Three Laboratories, First Affiliated Hospital of Anhui University of Chinese Medicine, No 117 Meishan Road, Shushan District, Hefei, China

## Abstract

Several previous studies have attempted to investigate the regulatory mechanisms underlying gene expression in ankylosing spondylitis (AS). However, the specific molecular pathways underlying this condition remain unclear. Previous research used next-generation RNA sequencing to identify a series of differentially expressed genes (DEGs) in peripheral blood mononuclear cells (PBMCs) when compared between patients with AS and healthy controls, thus implying that these DEGs may be related to AS. Furthermore, by screening these DEGS, it may be possible to facilitate clinical diagnosis and optimize treatment strategies. In order to test this hypothesis, we recruited 15 patients with AS and 15 healthy controls. We randomly selected five subjects from each group of patients for RNA sequencing analysis. Sequence reads were generated by an Illumina HiSeq2500 platform and mapped on to the human reference genome using HISAT2. We successfully identified 973 significant DEGs (*p* < 0.05) in PBMCs. When compared with controls, 644 of these genes were upregulated (with a fold change (FC) > 2) in AS patients and 329 were downregulated (FC < 0.5). Our analysis identified numerous genes related to immune response. Gene Ontology (GO) analysis indicated that these DEGs were significantly related to the positive regulation of epidermal growth factor-activated receptor activity, the positive regulation of the ERBB (erb-b2 receptor tyrosine kinase) signaling pathway, the differentiation of trophoblast giant cells, oxygen transport, immune-related pathways, and inflammation-related pathways. The DEGs were also closely related to the TNF and NF-*κ*B signaling pathways. Six DEGs were verified by quantitative real-time polymerase chain reaction (qRT-PCR). Receiver operating characteristic (ROC) curve analysis indicated that IL6 may represent a useful biomarker for diagnosing AS. The development of new biomarkers may help us to elucidate the specific mechanisms involved in the development and progression of AS.

## 1. Introduction

Ankylosing spondylitis (AS) is an immune-mediated chronic inflammatory form of arthritis and is characterized by chronic nonspecific inflammation and pathological bone formation, the latter representing a common clinical form of spondyloarthritis (SpA) [1]. The incidence of AS in China is approximately 0.3% and predominantly affects adults (mean age: 25 years; range: 15–35 years) [2]. Chronic inflammation of the spinal joints can lead to severe chronic pain and stiffness, ultimately leading to bone stiffness in the spine; this can also exert impact on several other systems [3]. AS is characterized by chronic progressive and refractory characteristics and can cause irreversible damage to the central axis of the spine; this results in the spine fusing with the sacroiliac joint, thus resulting in reduced spinal activity [4]. AS exerts serious effects on a patient's quality of life and is associated with a significant economic burden to society and families. Previous research suggested that this disease is highly correlated with the MHC (major histocompatibility complex) class I gene, HLA-B27 [5]. However, the specific cause of AS remains unclear, and therapeutic options for the treatment of AS remain inadequate. Over the last decade, new biological agents have been developed that have had a profound effect on the success rates of AS treatment. However, approximately 30% of patients fail to tolerate these drugs or experience differing degrees of adverse reactions [6]. Therefore, there is an urgent need to identify new biomarkers that may act as diagnostic or prognostic indicators for AS. The discovery of such biomarkers is likely to prove invaluable in the prevention, treatment, and control of this disease.

Previous researches involving the identification of molecular mechanisms and novel biomarkers associated with cancer, stroke, and diabetes have involved mRNA expression profiling performed by microarray analysis or high-throughput RNA sequencing [7–10]. Other studies have described the application of high-throughput technology for autoimmune diseases, such as systemic lupus erythematosus, autoimmune thyroid, and rheumatoid arthritis [11–14]. High-throughput methodology has already been used to study AS [15]; however, this previous study only focused on synovial tissue [15]. In the present study, we used RNA sequencing to construct a protein-coding gene regulation network in peripheral blood mononuclear cells (PBMCs) isolated from AS patients and healthy controls.

## 2. Material and Methods

### 2.1. Patients and Controls

Fifteen patients with AS were recruited from the Department of Rheumatology of the First Affiliated Hospital of Anhui University of Chinese Medicine. These patients were diagnosed by visiting staff according to the American College of Rheumatology (ACR) modified New York criteria [16, 17]. In addition, we also recruited 15 age- and sex-matched healthy subjects as controls. None of the patients or controls had any previous history of cardiovascular disease, diabetes, hepatitis, malignancy, or other autoimmune and inflammatory illnesses. The study was approved by the Medical Ethics Committee of the First Affiliated Hospital of Anhui University of Chinese Medicine (2015AH-20).

### 2.2. RNA Extraction and Sequencing

PBMCs were isolated from AS patients and healthy controls by Ficoll density gradient centrifugation and Lymphoprep (Stemcell, USA). Separated PBMCs were then lysed by a TRIzol Reagent (Invitrogen, USA) and stored at -80°C to await further processing. Total RNA was then extracted using a mirVana miRNA Isolation Kit (Ambion, Foster City, CA) in accordance with the manufacturer's instructions. A NanoDrop 2000 (Thermo Fisher Scientific, Waltham, MA) was used to evaluate the quantity of RNA, and an Agilent 2100 bioanalyzer (Agilent Technologies, Santa Clara, CA) was used to assess RNA quality. Purified libraries were prepared by Illumina TruSeq Stranded Total RNA Sample Preparation Kits (Illumina, San Diego, CA) in accordance with the manufacturer's instructions and quantified with a Qubit 2.0 Fluorometer (Life Technologies, Carlsbad, CA) and an Agilent 2100 bioanalyzer. cBot software was used to generate a cluster from libraries. The cluster was then sequenced on the Illumina HiSeq 2500 platform (San Diego, CA). All sequencing was performed by Origin-Biotech Inc. (Ao-Ji Bio-Tech, Shanghai, China).

### 2.3. Analysis of DEGs

FastQC was used to perform quality control assessments on raw sequence data arising from high-throughput sequencing pipelines (http://bioinformatics.babraham.ac.uk/projects/fastqc). Known Illumina TruSeq adapter sequences, poor reads, and ribosome RNA reads were trimmed by seqtk (https://github.com/lh3/seqtk) and mapped to the *Homo sapiens* reference genome (hg38) by HISAT2 software (version: 2.0.4) [18, 19]. Gene count was then analyzed by StringTie [19, 20] and normalized by the trimmed mean of M value (TMM) method [21]. We also determined the number of fragments per kilobase of transcript per million mapped reads (FPKM) using Perl script [22]. Differentially expressed genes (DEGs) were then determined by edgeR [23, 24] with a threshold of *p* < 0.05 and absolute values of log2(fold change) > 1 [7, 25, 26].

### 2.4. Functional Enrichment Analysis

Next, we aimed to gain a better understanding of the functionality of the DEGs identified. To do this, we used the R package (v 3.5.1) [27] and clusterProfiler to perform GO term enrichment analysis [28, 29] and KEGG [30] pathway analysis.

### 2.5. Protein-Protein-Interaction (PPI) Network Construction and Module Analysis

Next, we used the STRING tool [31] to map PPIs for all DEGs with a composite interaction score ≥ 0.4.

Cytoscape [32] was used to visualize the PPI network, and modules were filtered by the Molecular Complex Detection (MCOD) plug-in [33] using the following parameters: degree cut − off = 2, *k* − core = 2, node score cut − off = 0.2, and max depth = 100. Functional enrichment within each module was considered if the MCODE score was ≥4 and the node frequency was ≥10. GO and KEGG enrichment analysis for the DEGs within the four modules was performed in clusterProfiler.

### 2.6. Validation of DEGs

Quantitative real-time polymerase chain reaction (qRT-PCR) was used to verify the RNA sequencing data using *β*-actin as the internal control. Relative mRNA expression was calculated by the 2^-*ΔΔ*CT^ method [34]. In total, the expression levels of six genes were quantified (*TNFAIP3*, *IL1β*, *IL6*, *GPR55*, *CCR2*, and *CXCL5*). The primers used for qRT-PCR are shown in Table 1.

### 2.7. Statistical Analysis

Data are presented as the mean ± standard error of the mean. Data were analyzed using Student's *t*-test. *p* < 0.05 was considered to indicate a statistically significant difference. Student's *t*-test and ROC were analyzed by GraphPad Prism (version 8) and presented as the mean ± standard error of the mean.

## 3. Results

### 3.1. Screening of DEGs

**Figure 1:**
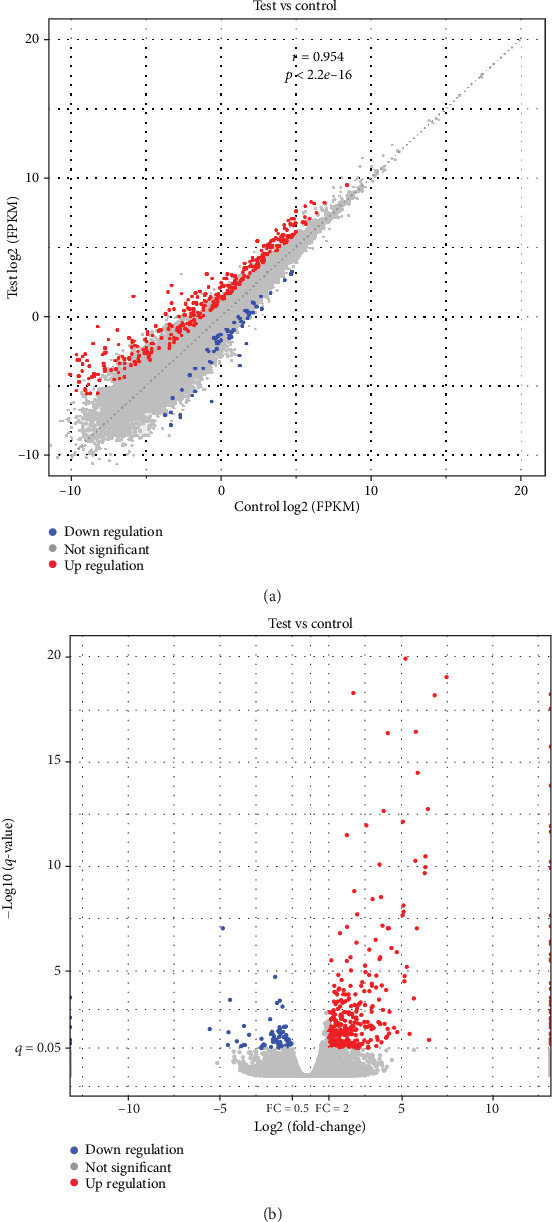
Volcano plot (a, blue and red indicate >twofold decreased and increased expression in AS, respectively) and scatter plot (b, blue and red indicate >twofold decreased and increased expression in AS, respectively) of DEGs between the AS and control groups. Gray indicates no significant difference.

**Figure 2:**
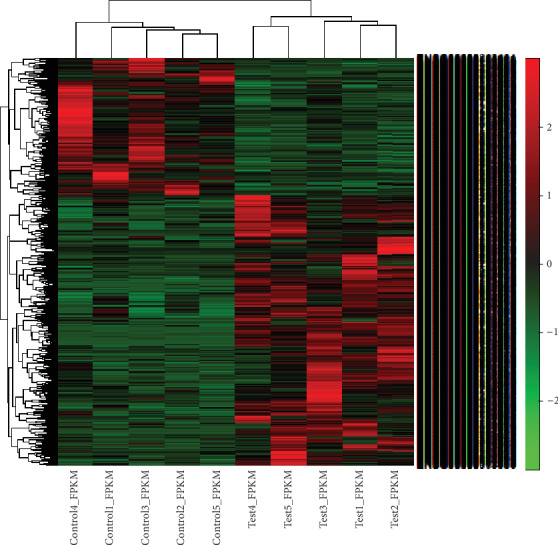
Hierarchical clustering of differentially expressed genes between the AS and control groups.

In total, 973 DEGs were identified between the AS and control groups, including 644 upregulated DEGs and 329 downregulated DEGs. These DEGs are represented by volcano and scatter plots in Figures 1(a) and 1(b), respectively.  Figure 2 shows the hierarchical clustering of DEGs. The most significant upregulated genes were T cell differentiation protein 2 (*MAL2*) and myomesin 2 (*MYOM*). The top 20 up- and downregulated DEGs are shown in Tables 2 and 3.

### 3.2. GO and Pathway Enrichment Analysis

**Figure 3:**
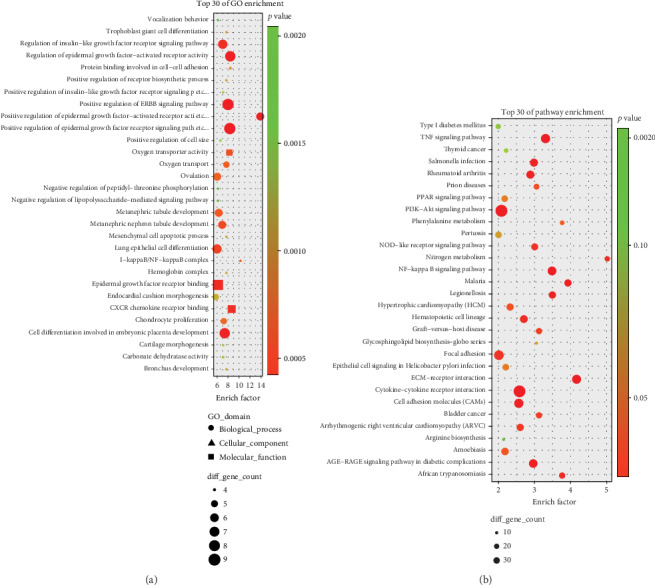
GO and pathway enrichment analysis results for DEGs. (a) Top 30 GO enrichment terms. (b) Top 30 pathway enrichment terms.

Next, we used clusterProfiler to perform GO enrichment analysis for the 973 DEGs. GO analysis placed the DEGs into 53 subclasses; Figure 3 shows the top 30 subclasses.

GO analysis revealed that the identified DEGs were predominantly associated with a range of biological processes, including positive regulation of the epidermal growth factor-activated receptor activity, positive regulation of the ERBB signaling pathway, the differentiation of trophoblast giant cells, and oxygen transport. In terms of cellular components, the NF-*κ*B complex was dominant. With regard to molecular function, our analyses identified epidermal growth factor receptor binding and CXCR chemokine receptor binding (Figure 3(a)).

Pathway analysis showed that the pathways most closely related to the DEGs were the TNF signaling pathway, the PI3K-Akt signaling pathway, cytokine-cytokine receptor interaction, and the NF-*κ*B signaling pathway (Figure 3(b)).

### 3.3. PPI Network Analysis

**Figure 4:**
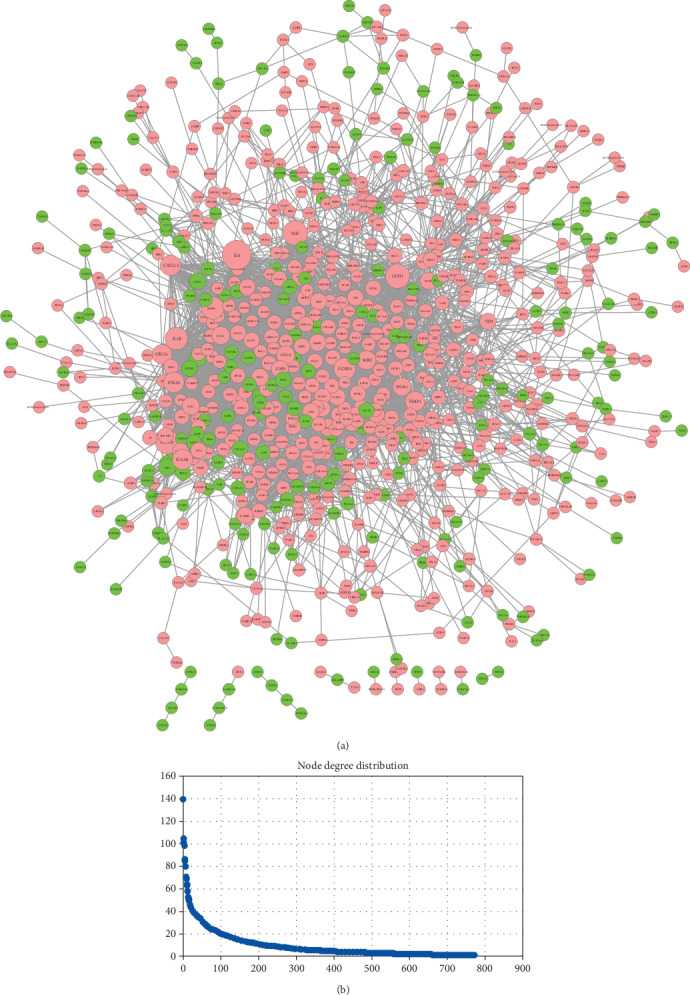
Visualization (a) and node degree distribution (b) of the PPI network. Node size is related to node degree; pink and green nodes denote up- and downregulated genes, respectively.

**Figure 5:**
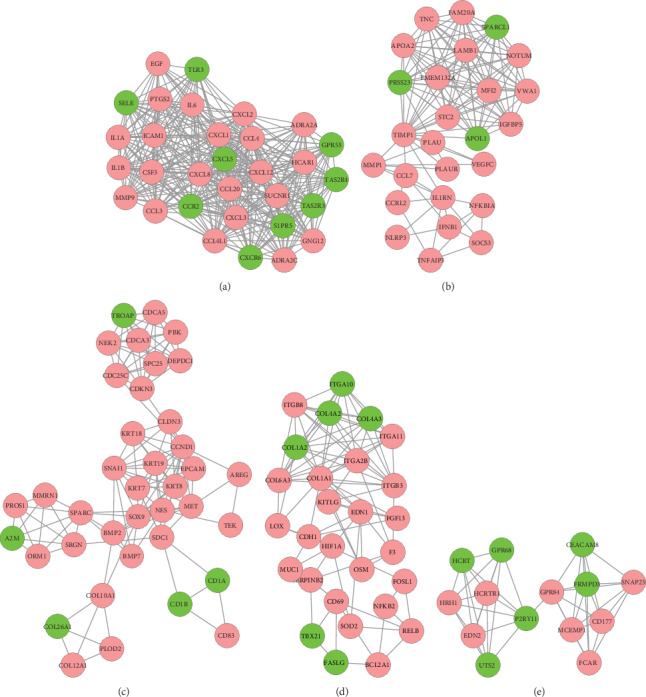
Significant module 1 (a), module 2 (b), module 3 (c), module 4 (d), and module 5 (e) subnet work of PPI networks. Pink and green nodes denote up- and downregulated genes, respectively. PPI: protein-protein interaction; DEGs: differentially expressed genes.

The DEGs identified in our analysis were then used to build a PPI network based on string databases (Figure 4(a)). The PPI network consisted of 768 nodes and 3824 edges. Network analysis showed that hundreds of genes were able to interact with 10 more other genes. In particular, IL6, EGF, and CDH1 were shown to interact with more than 100 genes; node distribution is shown in Figure 4(b). In total, 29 modules were identified by MCODE, operating with default criteria.  Table 4 lists these modules in descending order according to MCODE scores > 2. We selected five modules (modules 1, 2, 3, 4, and 5) with an MCODE score ≥ 3 and ≥10 nodes for module network visualization (Figure 5).

### 3.4. Verification of DEGs by qRT-PCR

**Figure 6:**
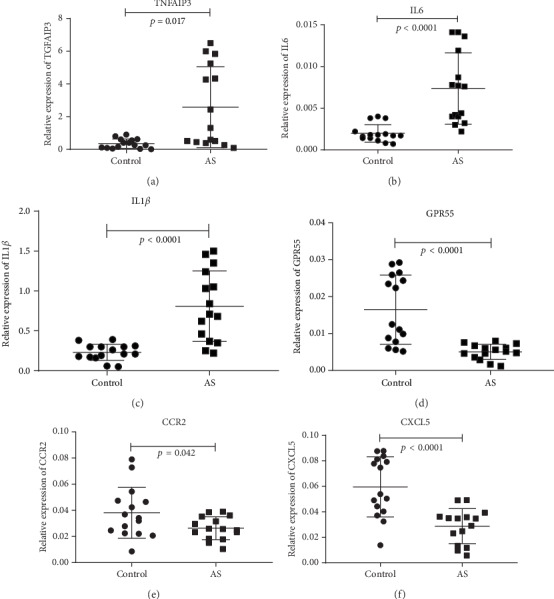
qRT-PCR verification of DEGs. Brain tissue expression of six genes was detected by qRT-PCR and is shown as expression fold changes. GAPDH was the internal control.

Compared with the control group, qRT-PCR detected significantly higher levels of expression for *TNFAIP3*, *IL1β*, and *IL6*, in the group of AS patients (Figure 6). AS patients also had lower expression levels of *GPR55*, *CCR2*, and CXCL5; this was consistent with the results derived from RNA sequencing, thus indicating that the qRT-PCR verification was reliable.

### 3.5. Receiver Operating Characteristic (ROC) Curve Analysis of Confirmed mRNAs in PBMCs

**Figure 7:**
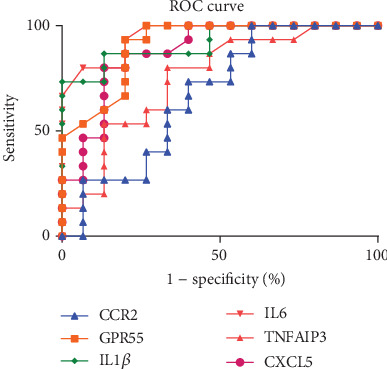
Visualization and details of the ROC curve.

ROC curve analysis was used to evaluate the potential diagnostic value of differentially expressed mRNAs that showed statistical significance. Our analysis showed that the levels of *TNFAIP3*, *IL1β*, *IL6*, *GPR55*, and *CXCL5* could discriminate between AS patients and controls. Analysis showed that *IL6* had the highest area under the curve (AUC: 0.9533; 95% confidence interval [CI]: 0.8872-1.019; *p* < 0.0001), followed by *IL1β* (AUC: 0.92; 95% CI: 0.8231-1.017; *p* < 0.0001), *GPR55* (AUC: 0.9089; 95% CI: 0.8041-1.014; *p* = 0.0001), *CXCL5* (AUC: 0.8778; 95% CI: 0.7508-1.005; *p* = 0.0004), and *TNFAIP3* (AUC: 0.7511; 95% CI: 0.5731-0.9291; *p* = 0.0191). Therefore, our analyses suggested that *IL6* (*p* < 0.0001) may be more valuable than the other four mRNAs as a biomarker for AS diagnosis (Figure 7).

## 4. Discussion

In the present study, we used high-throughput RNA sequencing to analyze protein-coding mRNA expression profiles in PBMCs isolated from AS patients and controls. We successfully identified 973 DEGs and then performed a range of analyses (GO, KEGG pathway, PPI, and PPI module) in an attempt to identify novel mechanisms for AS.

**Figure 8:**
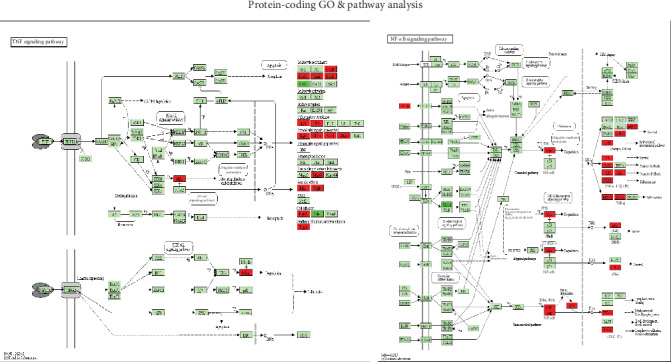
Significantly enriched KEGG pathways in the (a) TNF signaling pathway and (b) NF-*κ*B signaling pathway. Up-DEGs are marked in red. Down-DEGs are marked with dark green. The pictures were drawn with the KEGG Mapper (http://www.kegg.jp/kegg/tool/map_pathway2.html).

GO and pathway enrichment analysis revealed that numerous genes, associated with immune or inflammatory responses, may play a key role in AS. PPI results further demonstrated that the related genes have high degree. Our analysis also revealed that a number of GO terms were enriched, including positive regulation of acute inflammatory response (*p* = 3.170*e*‐03), acute inflammatory response (*p* = 7.623*e*‐05), positive regulation of inflammatory response (*p* = 1.247*e*‐03), regulation of acute inflammatory response (*p* = 8.621*e*‐03), regulation of inflammatory response (*p* = 1.988*e*‐05), inflammatory response (*p* = 3.196*e*‐09), negative regulation of inflammatory response (*p* = 3.462*e*‐02), immune system process (*p* = 4.903*e*‐03), T cell differentiation involved in immune response (*p* = 7.247*e*‐03), negative regulation of immune system process (*p* = 7.468*e*‐03), type 2 immune response (*p* = 8.962*e*‐03), regulation of immune system process (*p* = 9.386*e*‐03), immune system development (*p* = 1.139*e*‐02), response to immobilization stress (*p* = 1.267*e*‐02), regulation of type 2 immune response (*p* = 1.693*e*‐02), T cell activation involved in immune response (*p* = 1.993*e*‐02), leukocyte activation involved in immune response (*p* = 4.212*e*‐02), and cell activation involved in immune response (*p* = 4.525*e*‐02) [35–37]. We also identified several pathways that were enriched, including the NF-*κ*B signaling pathway (*p* = 3.288*e*‐05), TNF signaling pathway (*p* = 2.309*e*‐05), NOD-like receptor signaling pathway (*p* = 3.995*e*‐03), Salmonella infection (*p* = 7.149*e*‐04), rheumatoid arthritis (*p* = 9.785*e*‐04), cytokine-cytokine receptor interaction (*p* = 4.056*e*‐06), cell adhesion molecules (CAMs) (*p* = 5.342*e*‐04), focal adhesion (*p* = 4.886*e*‐03), and the chemokine signaling pathway (*p* = 1.994*e*‐02) [38–40]. Some clinical drugs have been reported to target TNFA (tumor necrosis factor alpha) [38, 39]; Figure 8(a) shows the results of our analysis related to the TNF signaling pathway. The present study demonstrated that AS patients are associated with cytokine disorders, excessive activation of NF-*κ*B signaling pathways, increased secretion of inflammatory cytokines, immune complex deposition, and immune-mediated inflammation [41]. Our screening results for the NF-*κ*B signaling pathway are shown in Figure 8(b). We hypothesize that AS could lead to immune disorders by regulating the differentiation and activation of T cells and other modes of immunization. Further research is now warranted to identify the specific function of these processes in AS.

In addition, we used qRT-PCR to identify several genes that are related to AS, including *TNFAIP3*, *IL1β*, *IL6*, *GPR55*, and *CXCL5*. The results of our qRT-PCR provided further verification that our high-throughput sequencing results were reliable. Our results also suggest that these genes could effectively distinguish between samples from AS patients and normal controls, thus providing a useful diagnostic tool.

## 5. Conclusions

Collectively, our findings provide clinically useful information relating to the mRNA profile of PBMCs in patients with AS. In addition, bioinformatic methodology was used to predict the potential functional roles of DEGs and explore their possible roles in the pathogenesis of AS. This information is likely to provide us with a better understanding of the pathogenic processes leading to AS. Future research should be aimed at investigating the specific biological functions and molecular mechanisms underlying the roles of these DEGs in the pathogenesis of AS.

## Figures and Tables

**Table 1:** 

Gene	Primer sequences	PCR product length (bp)
GAPDH	F: ACAACTTTGGTATCGTGGAAGG	101
	R: GCCATCACGCCACAGTTTC	
IL6	F: ACTCACCTCTTCAGAACGAATTG	149
	R: CCATCTTTGGAAGGTTCAGGTTG	
TGFAIP3	F: TCCTCAGGCTTTGTATTTGAGC	124
	R: TGTGTATCGGTGCATGGTTTTA	
IL1*β*	F: ATGATGGCTTATTACAGTGGCAA	132
	R: GTCGGAGATTCGTAGCTGGA	
CCR2	F: CCACATCTCGTTCTCGGTTTATC	88
	R: CAGGGAGCACCGTAATCATAATC	
CXCL5	F: GTTCCATCTCGCCATTCATGC	103
	R: GCGGCTATGACTGAGGAAGG	
GPR55	F: GAAAACCCTACAGTTTGCAGTCC	123
	R: GAGGTGGCAGCATAATCGGG	

**Table 2:** 

Gene name	Description	Test	Control	Log2FC	*p* value	Updown
ESRP1	Epithelial splicing regulatory protein 1	0.167880977	0.005006537	5.067482003	4.27*E*-16	Up
EFEMP1	EGF containing fibulin extracellular matrix protein 1	0.14670886	0.004273296	5.101462972	8.17*E*-12	Up
GPRC5A	G protein-coupled receptor class C group 5 member A	0.080655708	0.002331394	5.112511817	1.65*E*-11	Up
TENM3	Teneurin transmembrane protein 3	0.050467249	0.001415242	5.156226587	7.20*E*-08	Up
SYT7	Synaptotagmin 7	0.084086967	0.002342832	5.165557022	3.91*E*-08	Up
TBX2	T-box 2	0.06419033	0.001648608	5.283036021	1.28*E*-08	Up
ACOD1	Aconitate decarboxylase 1	0.207837546	0.004784204	5.441033669	8.91*E*-05	Up
KRT18	Keratin 18	4.672652849	0.102993604	5.503615208	1.74*E*-51	Up
CYP24A1	Cytochrome P450 family 24 subfamily A member 1	0.10699757	0.002094784	5.674632992	6.53*E*-07	Up
SLC7A2	Solute carrier family 7 member 2	0.091208901	0.001686833	5.75678535	4.33*E*-14	Up
IGFBP5	Insulin-like growth factor binding protein 5	0.306887146	0.005578492	5.78168917	1.25*E*-20	Up
TPD52L1	Tumor protein D52 like 1	0.054860254	0.0009603	5.836132086	1.31*E*-10	Up
ASS1	Argininosuccinate synthase 1	0.498890617	0.008439794	5.885371886	1.55*E*-18	Up
NPNT	Nephronectin	0.111594596	0.001448034	6.268027541	2.07*E*-13	Up
EMP2	Epithelial membrane protein 2	0.152045889	0.001923435	6.304677643	1.00*E*-13	Up
BCAM	Basal cell adhesion molecule (Lutheran blood group)	0.134791483	0.001698518	6.310308799	2.55*E*-14	Up
MYH14	Myosin heavy chain 14	0.113204752	0.00130165	6.442449138	9.53*E*-17	Up
GFRA1	GDNF family receptor alpha 1	0.143605056	0.001274655	6.815855972	1.86*E*-22	Up
KRT8	Keratin 8	2.717254001	0.01760557	7.26997371	1.22*E*-64	Up
MAL2	MAL, T cell differentiation protein 2 (gene/pseudogene)	0.599737909	0.003393226	7.465531038	1.67*E*-23	Up

**Table 3:** 

Gene name	Description	Test	Control	Log2FC	*p* value	Updown
MYOM2	Myomesin 2	0.083396198	2.342960807	-4.812207394	1.32*E*-10	Down
CCL14	C-C motif chemokine ligand 14	0.00720548	0.153612496	-4.414057235	7.88*E*-07	Down
PPARGC1A	PPARG coactivator 1 alpha	0.000951005	0.017674014	-4.216033153	0.001331584	Down
LUM	Lumican	0.003716002	0.067922087	-4.192057583	0.009079935	Down
FABP4	Fatty acid binding protein 4	0.011695583	0.173777145	-3.893202651	0.001508377	Down
SMIM17	Small integral membrane protein 17	0.013930292	0.201150581	-3.851978548	0.035328047	Down
FUT2	Fucosyltransferase 2	0.002487863	0.035674354	-3.841908383	0.019476857	Down
GPR20	G protein-coupled receptor 20	0.019006288	0.264915922	-3.800985884	0.000574724	Down
HCRT	Hypocretin neuropeptide precursor	0.029753156	0.396891741	-3.737630912	0.02520749	Down
COL1A2	Collagen type I alpha 2 chain	0.001038445	0.013406383	-3.690423438	0.007386025	Down
SCN5A	Sodium voltage-gated channel alpha subunit 5	0.001175881	0.014594963	-3.633657068	0.011433094	Down
SRPK3	SRSF protein kinase 3	0.002625025	0.030372663	-3.532370079	0.016367701	Down
CCDC144NL	Coiled-coil domain containing 144 family, N-terminal-like	0.004291893	0.04874123	-3.505456706	0.047045652	Down
CROCC2	Ciliary rootlet coiled-coil, rootletin family member 2	0.007272199	0.075178624	-3.369858992	0.000103433	Down
SLC18A1	Solute carrier family 18 member A1	0.004546282	0.045121799	-3.311065599	0.022179777	Down
OXCT2	3-Oxoacid CoA-transferase 2	0.017775459	0.174884534	-3.298443983	0.00139065	Down
PLSCR2	Phospholipid scramblase 2	0.015226302	0.124444283	-3.030862467	0.001263976	Down
BTN1A1	Butyrophilin subfamily 1 member A1	0.006703082	0.054223075	-3.016010489	0.038536757	Down
HYDIN	HYDIN, axonemal central pair apparatus protein	0.002046295	0.016428267	-3.005094491	0.021563849	Down
HSPB9	Heat shock protein family B (small) member 9	0.015111578	0.119979888	-2.989066346	0.028712686	Down

**Table 4:** 

Cluster	Score	Nodes	Edges	Node IDs
1	11.129	31	345	CXCL8, CSF3, TLR3, SELE, ICAM1, GNG12, GPR55, ADRA2C, SUCNR1, HCAR1, ADRA2A, TAS2R3, TAS2R4, S1PR5, CXCR6, CCL4L1, CXCL3, CXCL5, CCL3, CXCL12, CXCL2, PTGS2, CCR2, CCL20, CXCL1, EGF, CCL4, IL6, MMP9, IL1A, IL1*β*
2	4.654	26	121	TIMP1, TMEM132A, SPARCL1, VWA1, FAM20A, TNC, IGFBP5, STC2, MFI2, LAMB1, APOL1, APOA2, NOTUM, PRSS23, TNFAIP3, CCRL2, CCL7, NLRP3, IFNB1, SOCS3, NFKBIA, IL1RN, VEGFC, MMP1, PLAUR, PLAU
3	3.395	38	129	SNAI1, CLDN3, EPCAM, CD1A, CD83, CD1B, MMRN1, PROS1, ORM1, A2M, SRGN, SDC1, TROAP, DEPDC1, CDKN3, NEK2, CDCA5, SPC25, PBK, CDCA3, CDC25C, CCND1, KRT7, SOX9, COL26A1, COL12A1, PLOD2, COL10A1, BMP2, BMP7, SPARC, NES, TEK, AREG, MET, KRT19, KRT18, KRT8
4	3.25	28	91	OSM, MUC1, F3, FOSL1, SOD2, CD69, RELB, NFKB2, BCL2A1, FASLG, TBX21, SERPINB2, HIF1A, FGF13, KITLG, EDN1, CDH1, LOX, COL4A3, COL4A2, ITGA11, ITGB8, ITGA10, COL6A3, ITGA2B, ITGB3, COL1A2, COL1A1
5	3.071	14	43	HCRT, EDN2, UTS2, HRH1, P2RY11, GPR84, SNAP25, CD177, MCEMP1, CEACAM8, FCAR, FRMPD3, GPR68, HCRTR1
6	2.857	7	20	SOCS6, SPSB4, ASB1, FBXO27, FBXO17, KBTBD6, UBE3D
7	2.154	13	28	RORC, ULBP3, ULBP2, KLRC2, KIR3DL1, KLRB1, CALB1, ELAVL3, TBR1, MAP2, ENSG00000258947, FABP7, EOMES

## Data Availability

The RNA-seq data used to support the findings of this study are available from the corresponding author upon request.

## References

[1] Garcia-Montoya L., Gul H., Emery P. (2018). Recent advances in ankylosing spondylitis: understanding the disease and management. *F1000Research*.

[2] Machado P., Landewé R., Braun J. (2011). A stratified model for health outcomes in ankylosing spondylitis. *Annals of the Rheumatic Diseases*.

[3] Maksymowych W. P. (2010). Disease modification in ankylosing spondylitis. *Nature Reviews Rheumatology*.

[4] Bergman M., Lundholm A. (2018). Managing morbidity and treatment-related toxicity in patients with ankylosing spondylitis. *Rheumatology*.

[5] Tsui F. W., Tsui H. W., Akram A., Haroon N., Inman R. (2014). The genetic basis of ankylosing spondylitis: new insights into disease pathogenesis. *Application of Clinical Genetics*.

[6] Biggioggero M., Favalli E. G. (2014). Ten-year drug survival of anti-TNF agents in the treatment of inflammatory arthritides. *Drug Development Research*.

[7] Duan X., Gan J., Xu F. (2018). RNA sequencing for gene expression profiles in a rat model of middle cerebral artery occlusion. *BioMed Research International*.

[8] Neelankal John A., Ram R., Jiang F. X. (2018). RNA-seq analysis of islets to characterise the dedifferentiation in type 2 diabetes model mice db/db. *Endocrine Pathology*.

[9] Tian P., Liang C. (2018). Transcriptome profiling of cancer tissues in Chinese patients with gastric cancer by high-throughput sequencing. *Oncology Letters*.

[10] Beane J., Vick J., Schembri F. (2011). Characterizing the impact of smoking and lung cancer on the airway transcriptome using RNA-seq. *Cancer Prevention Research*.

[11] Odhams C. A., Cunninghame Graham D. S., Vyse T. J. (2017). Profiling RNA-seq at multiple resolutions markedly increases the number of causal eQTLs in autoimmune disease. *PLOS Genetics*.

[12] Zhang Q., Liang Y., Yuan H. (2019). Integrated analysis of lncRNA, miRNA and mRNA expression profiling in patients with systemic lupus erythematosus. *Archives of Medical Science*.

[13] Yi R., Yang L., Zeng S., Su Y. (2019). Different expression profile of mRNA and long noncoding RNA in autoimmune thyroid diseases patients. *Journal of Cellular Biochemistry*.

[14] Ouyang Q., Wu J., Jiang Z. (2017). Microarray expression profile of circular RNAs in peripheral blood mononuclear cells from rheumatoid arthritis patients. *Cellular Physiology and Biochemistry*.

[15] Thomas G. P., Duan R., Pettit A. R. (2013). Expression profiling in spondyloarthropathy synovial biopsies highlights changes in expression of inflammatory genes in conjunction with tissue remodelling genes. *BMC Musculoskeletal Disorders*.

[16] Calin A., van der Linden S. M., Cats A., Valkenburg H. A. (1985). Comment on article by van der Linden et al. Evaluation of diagnostic criteria for ankylosing spondylitis: a proposal for modification of the New York criteria.. *Arthritis & Rheumatism*.

[17] The H. S. G., Steven M. M., Van der Linden S. M., Cats A. (1985). Evaluation of diagnostic criteria for ankylosing spondylitis: a comparison of the Rome, New York and modified New York criteria in patients with a positive clinical history screening test for ankylosing spondylitis. *British Journal of Rheumatology*.

[18] Kim D., Langmead B., Salzberg S. L. (2015). HISAT: a fast spliced aligner with low memory requirements. *Nature Methods*.

[19] Pertea M., Kim D., Pertea G. M., Leek J. T., Salzberg S. L. (2016). Transcript-level expression analysis of RNA-seq experiments with HISAT, StringTie and Ballgown. *Nature Protocols*.

[20] Pertea M., Pertea G. M., Antonescu C. M., Chang T. C., Mendell J. T., Salzberg S. L. (2015). StringTie enables improved reconstruction of a transcriptome from RNA-seq reads. *Nature Biotechnology*.

[21] Robinson M. D., Oshlack A. (2010). A scaling normalization method for differential expression analysis of RNA-seq data. *Genome Biology*.

[22] Mortazavi A., Williams B. A., McCue K., Schaeffer L., Wold B. (2008). Mapping and quantifying mammalian transcriptomes by RNA-seq. *Nature Methods*.

[23] Nikolayeva O., Robinson M. D. (2014). edgeR for differential RNA-seq and ChIP-seq analysis: an application to stem cell biology. *Methods in Molecular Biology*.

[24] Robinson M. D., McCarthy D. J., Smyth G. K. (2009). edgeR: a bioconductor package for differential expression analysis of digital gene expression data. *Bioinformatics*.

[25] Benjamini Y., Drai D., Elmer G., Kafkafi N., Golani I. (2001). Controlling the false discovery rate in behavior genetics research. *Behavioural Brain Research*.

[26] Duan X., Han L., Peng D. (2019). High throughput mRNA sequencing reveals potential therapeutic targets of Tao-Hong-Si-Wu decoction in experimental middle cerebral artery occlusion. *Frontiers in Pharmacology*.

[27] Vrchoticky A. (2000). *R language definition*.

[28] Ashburner M., Ball C. A., Blake J. A. (2000). Gene Ontology: tool for the unification of biology. *Nature Genetics*.

[29] The Gene Ontology Consortium (2012). Gene Ontology annotations and resources. *Nucleic Acids Research*.

[30] Kanehisa M., Goto S. (2000). KEGG: Kyoto Encyclopedia of Genes and Genomes. *Nucleic Acids Research*.

[31] Szklarczyk D., Franceschini A., Kuhn M. (2010). The STRING database in 2011: functional interaction networks of proteins, globally integrated and scored. *Nucleic Acids Research*.

[32] Shannon P., Markiel A., Ozier O. (2003). Cytoscape: a software environment for integrated models of biomolecular interaction networks. *Genome Research*.

[33] Bader G. D., Hogue C. W. V. (2003). An automated method for finding molecular complexes in large protein interaction networks. *BMC Bioinformatics*.

[34] Livak K. J., Schmittgen T. D. (2001). Analysis of relative gene expression data using real-time quantitative PCR and the 2(-delta delta C(T)) method. *Methods*.

[35] Fiorillo M. T., Maragno M., Butler R., Dupuis M. L., Sorrentino R. (2000). CD8^+^ T-cell autoreactivity to an HLA-B27-restricted self-epitope correlates with ankylosing spondylitis. *Journal of Clinical Investigation*.

[36] Atagunduz P., Appel H., Kuon W. (2005). HLA-B27-restricted CD8^+^ T cell response to cartilage-derived self peptides in ankylosing spondylitis. *Arthritis and Rheumatism*.

[37] Hermann E., Meyer zum Büschenfelde K. H., Fleischer B., Yu D. T. Y. (1993). HLA-B27-restricted CD8 T cells derived from synovial fluids of patients with reactive arthritis and ankylosing spondylitis. *The Lancet*.

[38] Braun J., Davis J., Dougados M. (2006). First update of the international ASAS consensus statement for the use of anti-TNF agents in patients with ankylosing spondylitis. *Annals of the Rheumatic Diseases*.

[39] Baraliakos X., Listing J., Brandt J. (2005). Clinical response to discontinuation of anti-TNF therapy in patients with ankylosing spondylitis after 3 years of continuous treatment with infliximab. *Arthritis Research & Therapy*.

[40] Roozbehkia M., Mahmoudi M., Aletaha S. (2017). The potent suppressive effect of *β*-d-mannuronic acid (M2000) on molecular expression of the TLR/NF-kB signaling pathway in ankylosing spondylitis patients. *International Immunopharmacology*.

[41] Li F., Liu J., Lei W., Zhu F., Tan B., Zhang P. (2016). Xinfeng capsule improves hypercoagulative state by inhibiting miR-155/NF-*κ*B signaling pathway in patients with active ankylosing spondylitis. *Xi Bao Yu Fen Zi Mian Yi Xue Za Zhi*.

